# An “Omic” Overview of Fragile X Syndrome

**DOI:** 10.3390/biology10050433

**Published:** 2021-05-13

**Authors:** Olivier Dionne, François Corbin

**Affiliations:** Department of Biochemistry and Functional Genomics, Faculty of Medicine and Health Sciences, Université de Sherbrooke and Centre de Recherche du CHUS, CIUSSS de l’Estrie-CHUS, Sherbrooke, QC J1H 5H4, Canada; francois.corbin@usherbrooke.ca

**Keywords:** fragile X syndrome, FMRP, RNA-binding protein, physiopathology, genomic, transcriptomic, proteomic, metabolomic

## Abstract

**Simple Summary:**

Fragile X syndrome (FXS) is a neurodevelopmental disorder and remains the most frequent inherited cause of intellectual disability. Fragile X patients are at great risk to develop behavior problems including autism, anxiety, aggressivity and hyperactivity. FXS results from a mutation leading to the absence of a single protein, the fragile X mental retardation protein (FMRP). Most studies aiming to understand the physiopathology of FXS are centered on the effect of its absence on protein synthesis. However, besides protein synthesis regulation, FMRP has several other functions that need to be understood and could play a significant role in FXS. The goal of the present work is to review some of these functions and to put them into perspective in order to get a more comprehensive understanding of FXS physiopathology.

**Abstract:**

Fragile X syndrome (FXS) is a neurodevelopmental disorder associated with a wide range of cognitive, behavioral and medical problems. It arises from the silencing of the fragile X mental retardation 1 (FMR1) gene and, consequently, in the absence of its encoded protein, FMRP (fragile X mental retardation protein). FMRP is a ubiquitously expressed and multifunctional RNA-binding protein, primarily considered as a translational regulator. Pre-clinical studies of the past two decades have therefore focused on this function to relate FMRP’s absence to the molecular mechanisms underlying FXS physiopathology. Based on these data, successful pharmacological strategies were developed to rescue fragile X phenotype in animal models. Unfortunately, these results did not translate into humans as clinical trials using same therapeutic approaches did not reach the expected outcomes. These failures highlight the need to put into perspective the different functions of FMRP in order to get a more comprehensive understanding of FXS pathophysiology. This work presents a review of FMRP’s involvement on noteworthy molecular mechanisms that may ultimately contribute to various biochemical alterations composing the fragile X phenotype.

## 1. Introduction

**Figure 1:**
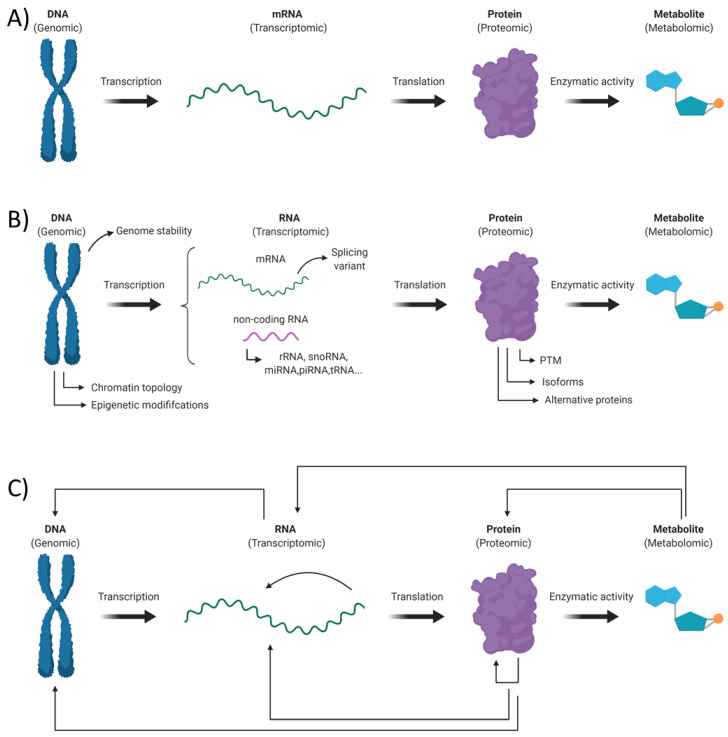
Schematic representation of the different “Omic” categories: (**A**) a simplistic “Omic” representation in which a gene encodes a single mRNA, which is further translated in a unique protein that can ultimately exert its biological activity, which in this example produces a metabolite. (**B**) A large diversity of biomolecules exists within each “Omic” category. (**C**) Schematization of the different interaction between component of each “Omic” categories.

The “Omic” term refers to the wide range of high throughput experimental approaches aiming to get a holistic depiction of the different biomolecules within a living organism. These techniques intend to get a better understanding of systems biology throughout a comprehensive overview of the genes (genomic), RNA (transcriptomic), proteins (proteomic) and metabolites (metabolomic) making up a cell or a tissue. However, when performing such studies, one should avoid the mistake of only getting a simplistic “Omic” portrait of its biological sample (Figure 1A). Indeed, an aware experimenter should take in consideration the large and inherent diversity within each class of biological molecules and the complex nature of the different interactions between them (Figure 1B,C).

Fragile X syndrome (FXS) is an X-linked neurodevelopmental disorder with an approximative prevalence of 1/4000 in males and 1/8000 in females [1]. FXS is the leading inherited cause of intellectual disability (ID) and the most prevalent monogenic cause of autism spectrum disorders (ASD). The severity of ID varies from one individual to another, being usually moderate to severe in men while it is most often mild to borderline in women. It is estimated that between 30% and 50% of patients with FXS will present ASD. Fragile X syndrome is also characterized by a broad spectrum of challenging behaviors (anxiety, aggression, self-injury, attention deficit disorder (ADHD, social withdrawal), physical (facial dysmorphia, macroorchidism, joints hypermobility) and self-limited medical problems (recurrent otitis media, seizures). Furthermore, all FXS-related comorbidities are found with variable penetrance, resulting in a large phenotypic heterogeneity across this population [2,3,4,5].

**Figure 2:**
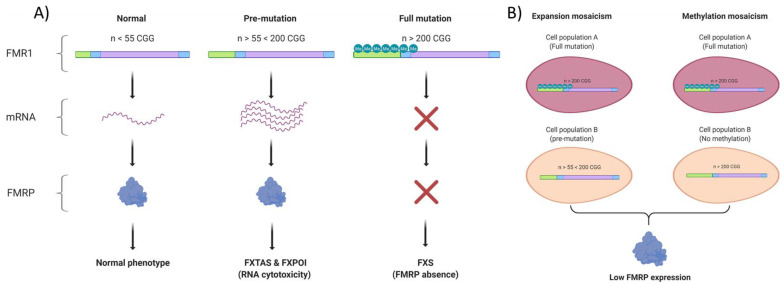
Genetics of fragile X syndrome: (**A**) CGG trinucleotide repeats are found between the promoter and the first exon of the FMR1 gene. A normal allele has fewer than 55 while a fully mutated one has more than 200. A full mutation leads to the silencing of the FMR1 gene throughout an epigenetic mechanism which is in part characterized by the methylation (Me) of the gene. A pre-mutation allele has between 55 and 200 CGG repeats and can lead to FXTAS (Fragile X Tremor and Ataxia Syndrome) or FXPOI (Fragile X Primary Ovarian Insufficiency). (**B**) Two types of mosaicism are observed in Fragile X. Expansion mosaicism is characterized by a cell population carrying a pre-mutation and another with a fully mutated and methylated allele. On the other hand, methylation mosaicism is characterized by two populations of fully mutated cells but only one of them carries a methylated allele. Both types of mosaicism result in low FMRP expression.

Fragile X syndrome typically originates from the dynamic expansion of CGG trinucleotide repeats found in the 5′ UTR of the FMR1 gene (Xq27.3) [6]. A full mutation allele possesses more than 200 repeats, which results in the transcriptional silencing of the gene throughout an epigenetic mechanism and ultimately in the loss of expression of the fragile X mental retardation protein (FMRP) [6,7]. Mosaicism is commonly observed in the fragile X population, with a prevalence ranging from 12–41% in males [8,9]. Repeats-size and methylation mosaicisms have been described in FXS and are both associated with a detectable FMRP expression, a higher IQ and milder behavioral problems [10,11,12,13] (Figure 2). X chromosome inactivation ratio in fragile X females (proportion of cells that have inactivated the fully mutated X chromosome) have also been correlated with a better prognosis [10].

FMRP is widely expressed across the body but to a higher degree in the brain and gonads [14,15]. FMRP possesses both nuclear localization and exportation signals, two Agenet domains, as well as two hnRNP homology domains (KH) and one arginine–glycine–glycine box (RGG) [16]. The last three domains provide FMRP RNA binding capacity. FMRP is able to recognize several RNA motifs such as pseudoknots’ structure, SoSLIP and G-quartets [17,18,19], as well as specific RNA sequences [20]. The RNA binding capacity of FMRP is central to its molecular function. Indeed, missense mutations in either KH domains impair FMRP ability to bind RNA and are associated with many typical FXS phenotypes, including ID, facial dysmorphia and macroorchidism [21,22]. Despite the fact that FMRP is involved in many stages of RNA metabolism, translational regulation remains its foremost function [23,24,25].

FMRP functions are essential for normal neurodevelopment, as illustrated by the many neuroanatomical defects harbored by fragile X patients. Indeed, abnormalities in white and grey matter volume of different brain regions have been observed in both young and adult patients [26,27,28]. Furthermore, post-mortem studies of fragile X brains have revealed an aberrant spine morphology, characterized by longer, thinner and denser dendritic spines [29,30]. Synaptic plasticity is impaired in the absence of FMRP and seems to be related to cortex hyperexcitability observed in FXS patients and several animal models [31,32,33]. This hyperexcitability seems to be the result of an enhanced long-term depression (LTD) and reduced long-term potentiation (LTP), the contribution of both mechanisms being different according to brain regions [34]. In some patients, the absence of FMRP may also affect other classic neurochemicals pathways, including the dopaminergic, cholinergic and serotonergic-mediated pathways [35,36].

**Figure 3:**
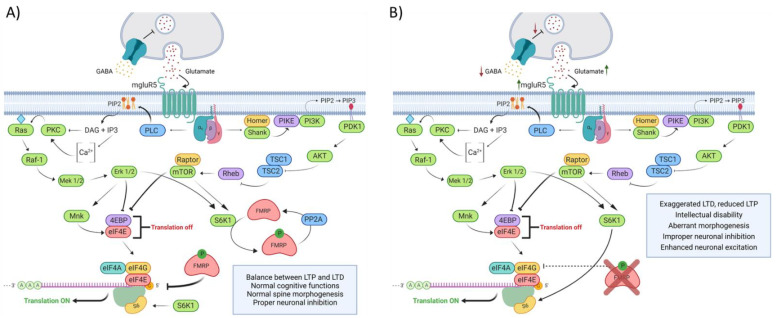
Schematization of FXS physiopathology, resulting from an imbalance between excitatory (glutamate) and inhibitory (GABA) neurotransmission. (**A**) Activation of GABA receptors at the presynaptic neurons inhibits the release of glutamate into the synaptic cleft. Glutamate stimulation of mGluR5 at the postsynaptic neuron leads to the activation of many downstream effectors, mainly the Ras/Raf/Mek/ERK and PI3K/AKT/mTOR signaling cascades. These pathways lead, among other things, to the translation of proteins which promote LTD and impair LTP. (**B**) GABA output is decreased in the absence of FMRP, resulting in an improper neuronal inhibition. The resulting enhanced glutamate release promotes the activation of mGluR5, which ultimately lead to an exaggerated LTD and a reduced LTP.

The preclinical research conducted over the past 20 years has mainly focused on the excitatory glutamate transmission mediated by group 1 (Gp1) metabotropic glutamate receptor (mGluR1 and mGluR5) and the γ-aminobutyric acid (GABA) related inhibition to link the lack of FMRP to the mechanisms underlaying FXS physiopathology (Figure 3). The hypothesis based upon those observations are centered around FMRP’s ability to regulate the translation of its targeted mRNA. In this conjecture, FMRP would act as a chief regulator of Gp1 mGluR signaling by mediating the translation of proteins involved in its signal transduction and by functionally opposing the pro-translational effects of Gp1 mGluR stimulation [24]. The upregulation of Gp1 mGluR signaling results in aberrant LTD and LTP found in the *Fmr1* KO mouse hippocampus [37,38]. Deficits in GABAergic inhibition are also found in the FXS mouse model. Indeed, FMRP has been shown to regulate the translation of some GABA receptors subunits and many enzymes involved in GABA metabolism and transport [39,40,41,42]. Taken together, those alterations are thought to create an imbalance between excitatory and inhibitory neurotransmission and are believed to underline the cognitive and behavioral phenotypes observed in FXS.

This model of FXS physiopathology has been corroborated over the years by a plethora of animal model-driven studies. Indeed, the restoration of GABA and glutamate neurotransmission in *Fmr1* KO mice have repeatedly been associated with the rescue of FX biochemical and behavioral phenotypes [43,44,45,46,47,48,49,50]. Unfortunately, the hype carried out by those preclinical investigations did not translate into human, as clinical trials aimed to inhibit mGluR5 or to enhance the GABAergic system did not completely fulfill the expected outcomes [51,52,53,54,55,56,57,58,59]. Lessons can be learned from this inability to translate the success generated in the mouse model to the fragile X patient. Firstly, a better understanding of human physiopathology is needed. Given the accumulation of evidence regarding the limitation of the KO mouse to fully replicate human phenotype, studies aiming to fulfill this task must focus on human subjects and models [60,61]. Secondly, one should “think outside of the ribosome” when searching for new mechanisms underlaying FXS physiopathology. The bulk of the research addressing this topic has solely considered FMRP as a translational regulator. The assumption that FXS is merely a disease of erroneous translation compose an oversimplification of the broad diversity of biochemicals and cellular alterations composing the fragile X phenotype. Finally, one should always keep in mind the large phenotypic heterogeneity found across the FXS population.

**Figure 4:**
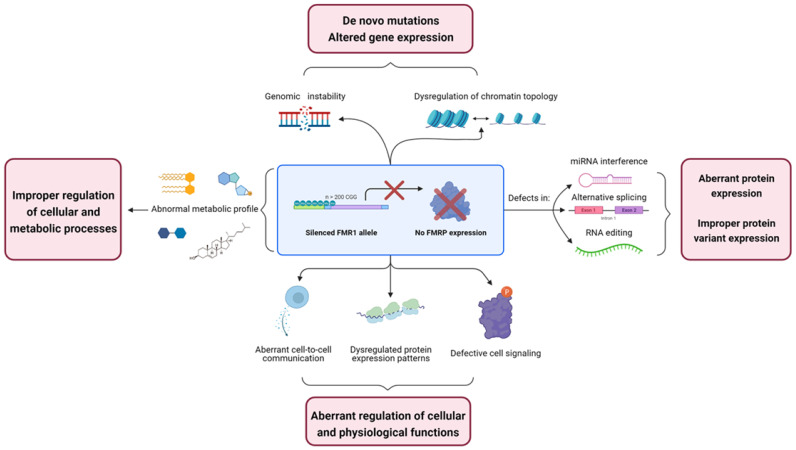
Summary of some of the various biochemicals alterations composing the fragile X phenotype on each “Omic” category.

This work proposes an overview of some of the numerous molecular mechanisms in which FMRP is involved and the multidimensional nature of the biochemicals defects found in fragile X (Figure 4). This will be accomplished by reviewing some of the different alteration observable in FXS in an “Omic-dependent” manner. Furthermore, this review will focus, as much as possible, on research conducted in human subjects or models.

## 2. Genomic Alterations in Fragile X Syndrome

Even though the vast majority is cytoplasmic, a certain proportion of FMRP is located in the nucleus. Some isoforms in which the nuclear export signal is spliced out are indeed found predominantly in this cellular compartment [62,63,64]. Those observations initially raised the hypothesis that FMRP may have actual nuclear function.

### 2.1. FMRP Is Involved in Genomic Stability Maintenance

One of the first reports of FMRP genomic functions came from the study of Alpatov et al., in which they brought strong evidence of FMRP implication in DNA damage response (DDR) [65]. They discovered that FMRP, through its Agenet domains, binds the chromatin through the methylated tails of histones H3. Mouse embryonic fibroblasts (MEF) treated with replication stress agent showed a FMRP-dependent induction of histone H2A variant H2A.X phosphorylation (γH2A.X), a core component of the DNA repair complex [66]. They also showed that under these conditions, more FMRP is located in the nucleus and that some of it colocalize with γH2A.X. FMRP transitory expression can rescue γH2A.X induction in HeLa and MEF cells, but mutations impairing Agenet domain binding to the chromatin prove to be less efficient in the matter. The precise role of FMRP in DDR remains, nonetheless, to be fully elucidated.

Evidence of the drosophila homolog of FMRP (dFMRP) involvement in the Piwi-interacting RNA (piRNA) pathway have also been reported [67,68]. The piRNA pathway is involved in the transcriptional silencing and enzymatic degradation of transposable elements, which have the ability to replicate and insert into new loci [69]. The piRNA mediated inhibition is crucial for the maintenance of genome integrity, as transposition events can lead to DNA breaks and improper recombination.

Taken together, those studies point toward a role for FMRP in the maintenance of genomic stability, which further suggests that fragile X patients may be subject to an abnormal increase of mutations caused by an accumulation of DNA damage and improper transposition events. This phenomenon can have a deleterious effect on long-lasting cells such as neurons and potentially have repercussion on the wider nervous circuit in which affected neurons are connected.

### 2.2. Alteration of Chromatin Topology in FXS

Regulation of the genome three-dimensional structure, also known as chromatin topology, is crucial for the spatiotemporal control of gene expression. This tightly regulated mechanism is mediated in part by post-translational modifications (PTM) of histones. These modifications dictate histone’s interactions with other proteins and modulate the reorganization of chromatin topology, two mechanisms which can modulate transcription. Indeed, some histone PTM exacerbate a repressive control over gene expression, while others promote it [70].

Many chromatin modifier enzymes are encoded by FMRP mRNA targets [71]. Reports of widespread dysregulation of histone PTM have been described in the mouse model of FXS, thus supporting this observation. Indeed, Korb et al. have monitored an increase of several histone modifications associated with an active chromatin topology in cortex neurons of *Fmr1* KO mice [72]. Ensuing RNA-seq analysis consequently revealed more upregulated genes among all dysregulated transcripts. Furthermore, they showed that pharmacological inhibition of the transcription factor Brd4 (a protein that binds to acetylated histones and promotes transcription [73]) normalize spine morphology, aberrant behavior and partially rescue gene expression in the same model [72]. A more recent report has described an overall increase in H3 lysine 36 trimethylation in the hippocampus of the *Fmr1* KO mouse [74], a PTM also associated with active gene expression [70].

Those studies highlighted the fact that the loss of FMRP promotes changes in chromatin topology which will result in a dysregulation of gene expression. Mutations in chromatin remodeling genes have also been repeatedly associated with neurodevelopmental disorders such as autism and ID [75,76,77]. Taken as a whole, those observations suggest that the alteration in histone PTM observed in the *Fmr1* KO mouse may cause aberrant gene expression, which in turn, underlie key fragile X phenotypes.

## 3. Transcriptomic Alterations in Fragile X Syndrome

By its capacity to bind both RNA and proteins, FMRP acts as an adapter to facilitate the interaction between those two classes of biomolecules. This pivotal function allows FMRP to mediate various RNA processing mechanisms other than its traditional ability to regulate translation by ribosome stalling or eIF4E sequestration (formation of a protein complex comprising FMRP and eIF4E resulting in the inhibition of translation initiation) [20].

### 3.1. FMRP Mediates Micro RNA-Related Interference

MicroRNAs (miRNAs) are 19–24 nucleotides long non-coding RNAs involves in post-transcriptional regulation of approximately 30% of human genes [78]. Upon transcription, newly synthesized primary miRNAs are processed into a partially unpaired stem-loop precursor (pre-miRNA) by the nuclear ribonuclease Drosha. Pre-miRNAs are then exported through exportin 5 to the cytoplasm, where they are further processed by the ribonuclease Dicer into double-stranded RNA. The functional strand of this RNA duplex makes up the mature miRNA and is loaded into the RISC complex (RNA induced silencing complex) through its association with members of the Argonaute (AGO) protein family. From there, miRNA-containing RISC complex binds to complementary mRNA and either promotes its degradation or inhibits its translation [79,80].

FMRP is a well-established effector of the miRNA pathway. Indeed, FMRP is found in RISC and directly interacts with different components of those ribonucleoprotein (RNP) complexes, including AGO1, AGO2 and Dicer and binds to both mature and pre-miRNAs [81,82,83,84,85]. FMRP binding to miRNAs is mediated by its KH domains and is believed to favor miRNA annealing to their complementary mRNA [83]. As such, in vitro experiments have shown that the presence of FMRP in RISC complex can both promote and inhibit miRNAs-related interference. The function of FMRP in that miRNA-containing RNP complex is modulated by many factors, including the affinity of FMRP to the targeted mRNA and protein-protein interactions [81,83,86].

First evidence of the involvement of the miRNA pathway into fragile X physiopathology was brought by Jin et al. [82]. In this study, they showed that a downregulation of AGO1 alters FMRP-related translational control and promotes certain FXS phenotypes. Moreover, this work establishes FXS as the first neurologic disease associated with alteration in the miRNA pathway. Since then, an accumulation of experimental evidence has linked such deficit to the absence of FMRP and, consequently, to mechanisms underlying fragile X physiopathology. For example, the miR-125b mediated translational repression of NR2A (a sub-unit of the glutamate receptor NMDA) is FMRP-dependent and regulates spines morphogenesis [87]. Defects in axon guidance can also be linked to aberrant miRNA regulation in FXS. Indeed, Halevy et al. have used induced pluripotent stem cells derived from fragile X subjects’ fibroblasts to associate a downregulation of hsa-miR-382 to this neuronal alteration [88]. FMRP was also shown to modulate the miR-125a mediated interference of PSD-95, a protein involved in post-synaptic AMPA receptors endocytosis and consequently, in synaptic plasticity regulation [89,90]. PSD-95 and miR-125a are respectively found up and downregulated in synaptoneurosomes of *Fmr1* KO mice. Even more interestingly, a recent miRNA profiling performed in two independent cohorts has identified an increase of miR-125a in urine of fragile X boys [91].

The miRNA pathway represents a third mechanism, with ribosome stalling and eIF4E sequestration, through which FMR exerts its regulatory effect on translation [20]. Correction of an improper miRNA interference therefore represents a potent therapeutic approach to rectify FXS biochemical alterations [79,92]. Furthermore, as shown by Putkonen et al. [91], circulatory miRNA may also constitute a great source of peripheral biomarkers that can be used as quantitative and objective tools by clinicians [93].

### 3.2. Alternative Splicing and FMRP

The first insight of FMRP involvement in alternative splicing came from the study of Didiot et al. [94] in which they showed that FMRP affects alternative splicing of its own mRNA. Indeed, they showed that FMRP bind its own mRNA trough two G-quartets located closely to a known alternative spliced site in exon 15. Consequently, they observed that both overexpression and silencing of FMRP leads to aberrant FMR1 splicing pattern [94]. Further evidence of FMRP implication into a more widespread regulation of alternative splicing comes from studies conducted in drosophila. Indeed, the knock-down of dFMRP leads to the deregulation of more than 100 splicing events [95]. Moreover, only a handful of dFMRP mRNA targets have seen their splicing pattern influenced by this genetic manipulation [96]. Those observations were recently transposed into the mouse model of fragile X. Indeed, Shah et al. [74] reported a reduction of approximately 30% of exon skipping event in *Fmr1* KO mice hippocampus. Furthermore, many of the genes found with defective splicing are linked to neuronal transmission and autism [74]. FMRP is also known to interact with various proteins involved in pre-mRNA alternative splicing, one of which being the splicing factor RBM14 [97,98]. Together, FMRP and RBM14 modulate the splicing of Tau and Protrudin, two genes involved in neuronal cells differentiation and dendritic spines growth. The improper splicing pattern of those two genes observed in the hippocampus of *Fmr1* KO mice therefore suggests that FMRP involvement in the regulation of pre-mRNA splicing can contribute to FXS physiopathology [98].

Alternative splicing is one of the most relevant mechanisms involved in the temporal and cell-specific regulation of gene expression and in the promotion of the proteome functional diversity. Maintenance of its homeostasis is thus mandatory to ensure appropriate cell function, especially in neural tissue where it participates in virtually all facets of neurons activity [99]. Despites mounting evidence regarding FMRP role in alternative splicing, the exact functions of FMRP in this molecular mechanism remain to be fully elucidated. More importantly, no study has yet corroborated those observations in specimens derived from fragile X patients.

### 3.3. FMRP Absence May Lead to Defect in RNA Editing

The ADAR (adenosine deaminase acting on RNA) family of enzymes catalyzes the post-transcriptional modification of adenosine to inosine in double-strand pre-mRNA. Inosine is interpreted as guanosine by the translational and splicing machinery. As such, A to I editing in mRNA may lead to variable amino acids incorporation into corresponding proteins and contribute to the regulation of its alternative splicing. In other words, RNA editing represents another mechanism by which different protein variants can emanate from one genomic entity. Furthermore, proper ADAR editing is believed to be crucial for normal neuronal functions, as many mRNA edited by those enzymes encode ion channels, neurotransmitter receptors and protein involved in synaptic transmission [100].

The initial observation of FMRP presence into ADAR-containing RNP complexes raised the possibility that FMRP may act has an RNA editing mediator [101]. Co-immunoprecipitation experiments report a direct interaction between FMRP and ADAR2, a partnership that was shown to inhibit ADAR2 RNA editing activity [102,103]. FMRP-dependent dysregulation of RNA editing was further observed in various models of FXS. Indeed, both FMRP silencing and mutations impairing KH domains RNA binding have proved to increase ADAR activity and alter editing for some of the mRNA studied [101,102,103]. These transcripts, which include the calcium channel Cav1.3 and glutamate receptor subunits GluK2, GluA2 and GluA4, are involved in synaptic modulation, thereby suggesting that RNA editing impairment may significantly contribute to FXS physiopathology [102,103]. However, those studies only considered the editing pattern of a preselected pool of mRNAs. A transcriptome wide characterization of RNA editing defects in fragile eventually clarify involvement of FMRP in RNA editing and the contribution of this mechanism to FXS phenotype.

## 4. Proteomics Alterations in Fragile X Syndrome

Expression patterns of numerous proteins are known to be deregulated in FXS. Those proteins exhibit a wide range of biological and cellular functions, ranging from receptors, enzymes, translation factors and more. The expected pathophysiological consequences of FMRP’s absence should therefore be as various as the nature of the proteins found improperly expressed in FXS.

### 4.1. Rate of Protein Synthesis Alteration: A Still Misunderstood Hallmark of FXS

Changes in the rate of protein synthesis remains a focal point of fragile X physiopathology given the general agreement regarding FMRP as a translational regulator. The first reported evidence of FMRP’s ability to regulate translation came from studies conducted in rabbit reticulocyte lysate, in which it was shown to inhibit the general translational rate in a dose-dependent manner [104,105]. Since then, a plethora of studies have employed a variety of FXS animal models, in combination with a profusion of experimental workflow, to firmly establish the relation between the absence of FMRP and an increased rate of protein synthesis [106,107,108].

Most of the work addressing this issue has been performed in order to conceptualize the mGluR theory, and consequently, in hippocampal slices prepared from *Fmr1* KO mice. This experimental approach, which allows the combination of electrophysiological and metabolic measurements, has greatly contributed to the advancement of knowledge regarding synaptic and biochemical defects underlying the absence of FMRP and supports the development of various preclinical studies [43,109]. In fact, rescue of the fragile X phenotype in *Fmr1* KO mice following targeted pharmacological treatments has repeatedly been associated with a normalization of the aberrant protein synthesis [44,45,47,110,111]. Such reports strengthen the role of FMRP in the maintenance of translational homeostasis to ensure normal neurologic and behavioral phenotypes and further establish the rate of protein synthesis alteration as one of the most potent monitoring biomarkers for FXS.

Despite being one of the most prominent and studied alterations in animal models, only a limited number of reports have studied protein synthesis defects in human, mainly due to limitations regarding sample nature and accessibility. Two studies have used fibroblasts obtained from a skin biopsy of FXS patients to monitor an increase in translational rate in fragile X derived cells [112,113]. Another substantial contribution concerning this issue came from two studies of the Beebe Smith group, in which positron emission tomography was used to measure a decrease of the in vivo integration of [^11^C] leucine into nascent brain proteins of 15 FXS patients under propofol sedation and a similar, but not significative, trend of perturbation in 9 FXS patients under dexmedetomidine anesthesia [114,115]. Finally, we recently reported that the rate of protein synthesis is also decreased in freshly extracted peripheral blood mononuclear cells (PBMCs) of FXS patients (Dionne et al., 2021, accepted, DOI: 10.1371/journal.pone.0251367). These studies undoubtedly shown that the perturbation in translational homeostasis found in animal models is replicated in fragile X patients. Furthermore, all these studies made the common observation that some FXS patients displayed a rate of protein synthesis within control range, thereby suggesting that any therapeutic intervention aimed at restoring translational homeostasis may not be beneficial for all of them. The tissue-specific trend of perturbation and variability observed into the FXS population seemingly demonstrates that the protein synthesis defects inherent to the absence of FMRP is more nuanced and complex in humans than in mice. Indeed, protein synthesis in KO mice was constantly shown to be solely upregulated in all tissue or cell types tested so far [113,116,117].

These observations clearly demonstrated that defects regarding protein synthesis are still misunderstood in humans, and that several questions remain to be addressed to clarify the situation. The first one would be to establish if normalization of the aberrant protein synthesis rate can be achieved by a targeted pharmacological treatment, and further confirm the usefulness of the rate of protein synthesis measurement as a monitoring biomarker for FXS. The development of such an objective and quantitative tool would represent a major step forward for any future FXS clinical trials [118]. Another substantial advancement would be to identify the proteins that are dysregulated during the timeframe that protein synthesis rate measurement is conducted. The same experimental approach could be used to study how FXS cells respond to different stimuli. Such experiments could provide valuable information on molecular mechanism underlying fragile X physiopathology and yield a relevant source of peripheral biomarkers [116].

### 4.2. Cell Signaling Defects in FXS

Cell signaling is a complex communication network that governs cell fundamental mechanisms and coordinate their activity. Through this process, extracellular signals activate specific cell surface receptors before being transduced by signaling cascades into a wide variety of intracellular responses. The Ras/Raf/MEK/ERK and the PI3K/AKT/mTOR pathways are two major actors of those cell signaling transduction mechanisms. Indeed, those ubiquitous pathways are pivotal for a broad range of cellular (cell proliferation and differentiation, transcription and translation regulation) and physiological (metabolism, synaptic plasticity, development) processes [119,120].

FMRP, through its ability to regulate the translation of its targeted mRNA, acts as a chief mediator of the two aforementioned pathways. Indeed, analyses conducted in different mouse brain regions and HEK293 cells have shown that FMRP binds the mRNA of various effectors and regulators of those signaling cascades [25,71,121]. Consequently, a basal hyperphosphorylation of ERK and AKT (reflecting the hyperactivation of their respective pathways) as well as a reduced ERK activation following Gp1 mGLUR stimulation were observed in the hippocampus and cortex of *Fmr1* KO mice [122,123,124,125]. Those alterations can be directly linked to exaggerated mGluR5 signaling and contribute to the aberrant synaptic phenotype and dysregulated protein synthesis rate observed in this model. As such, the normalization of ERK and AKT phosphorylation status in KO mice have frequently been associated with the correction of the improper translational rate, LTD and behavioral phenotypes; thereby establishing measurements of ERK and AKT activation status as one of the most potent biomarkers of therapeutic efficiency for FXS [44,45,109,110,126].

The hyperactivation of the Ras/Raf/MEK/ERK and PI3K/AKT/mTOR pathways found in the brain of KO mice is also observable in a variety of human samples. Indeed, an increase in the phosphorylation of MEK, ERK, AKT and mTOR have been described in post-mortem brains of FX patients [127,128]. These molecular alterations are also replicated in more accessible and less invasive tissues, such as fibroblasts, PBMCs and blood platelets [112,127,129]. Most notably, our research group showed that the phosphorylation of ERK and AKT measured in blood platelets is correlated with the IQ of FXS patients [129]. Furthermore, a three-month lovastatin treatment corrected the exaggerated ERK overactivation in platelets and has been associated with a clinical improvement [130]. However, no study has yet shown that a reduction of the AKT pathway hyperactivation is achievable following a targeted treatment in human trials.

Taken together, these observations clearly illustrate the relevance of using cell signaling alterations in FXS as biomarkers of therapeutic efficiency in human trials. In addition, it could also be interesting to study whether the signaling defects observed outside of neurons can contribute to the physiopathology of FXS. Given the multifunctional nature of these signaling pathways and the fact that their hyperactivation is replicated in peripheral cells, it is very likely that their deregulation in non-neuronal tissues could contribute, to some extent, to a number of extra-neuronal manifestations or symptoms.

### 4.3. The Matrix Metalloproteinase-9 Involvement in FXS Physiopathology

The matrix metalloproteinase-9 (MMP9) is a zinc-dependent endopeptidase which targets many components of the extracellular matrix (ECM). In neuronal tissue, MMP9 enzymatic activity towards the perineuronal net (a network of loosely organized ECM components that supports several synaptic processes [131,132]) promotes dendritic spines elongation and regulates synaptic plasticity [133,134,135,136].

Colocalization studies conducted in mouse neurons have shown that MMP9 mRNA is bound by FMRP and subjected to its translational control [137]. Consequently, an increase in MMP9 expression has been reported in the frontal cortex and hippocampus of FXS patients and *Fmr1* KO mice [137]. Furthermore, genetic deletion of MMP9 in *Fmr1* KO mice rescued several FXS phenotypes, including the aberrant spine morphogenesis and synaptic plasticity [138]. Those observations prompted the uses of minocycline, an MMP9 inhibitor, as a targeted treatment for FXS [139].

Minocycline treatments were shown to successfully reduce MMP9 activity, reverse the improper spine morphogenesis and improve anxiolytic behavior and locomotor activity in *Fmr1* KO mice [140,141]. During clinical trials, minocycline mildly improved FXS patient’s behavior, with a more pronounced effect on irritability and anxiety [142,143]. Dziembowska et al., further reported that the increased MMP9 plasma activity is reduced in some FXS patients following a three-month minocycline treatment [144]. Even though they did not observe any correlation between MMP9 inhibition and global clinical improvement, their results strongly suggest that plasma measurement of MMP9 can be used as a monitoring biomarker for FXS. A thorough validation within a larger FXS cohort is, however, mandatory to confirm that statement.

Those reports clearly demonstrated that MMP9 is a key contributor to FXS physiopathology. However, the precise mechanism by which MMP9 activity contributes to fragile X phenotype remains to be fully elucidated. Despites this, measurement of MMP9 proteolytic activity remains one of the promising biomarkers of FXS, as shown by the fact that the MMP9 dysregulation monitored in KO mice and post-mortem patient brains is replicated in FXS blood.

### 4.4. Aberrant Cytokines Profile: A Sign of Immune Dysfunction in FXS?

Cytokines are crucial mediators of cell signaling events occurring within the immune system and are secreted in response to various stimuli. A wide variety of extra-immune cells, including neurons and microglia, express cytokines and their respective receptors. Cytokines are consequently involved in the regulation of several neuronal functions, in both developing and mature brains, as well as in the nervous system response to infections and injuries. Such processes are important for normal brain operation, since they can modulate cognition and emotional processing [145].

Very little is known about a possible immune dysfunction in FXS since only a handful of exploratory studies have been reported. As such, an in situ comparative transcriptomic analysis has shown that dysregulated transcript from *Fmr1* KO mouse embryos hippocampus and cortex primarily harbor an immunological signature [146]. A further study observed a decrease in IL-6 and TNF-α mRNA expression in *Fmr1* KO mouse adult hippocampus [147]. Moreover, FXS PBMCs show an increase in the production of pro-inflammatory cytokines IL-6 and IL-12p40 after being conjointly stimulated with lipopolysaccharides (LPS) and DHPG, a Gp1 mGluR agonist. Interestingly, FXS leukocytes did not display any alteration in cytokine production when solely stimulated with LPS [148]. However, two studies have shown that circulating levels of several pro-inflammatory cytokines are actually decreased in FXS serum and plasma [149,150]. Despites being somewhat contradictory, those reports strongly suggest the existence of immune dysfunctions in FXS. This statement is supported by a recent systemic analysis of the medical diagnoses performed on 5736 FXS patients. Indeed, Yu et al. found a higher prevalence of various infectious diseases and an underrepresentation of autoimmune disorders in the FXS population [151].

An alteration in cytokines secretion or response could contribute to several FXS phenotypes, as it might increase the patient’s sensitivity to neurological damage induced by environmental, pathological or xenobiotic exposure. However, more detailed studies are needed to better characterize the potential immune dysfunctions found in FXS and the mechanism by which the absence of FMRP leads to such impairments.

## 5. Metabolomic Alterations in Fragile X Syndrome

Many studies have associated an aberrant metabolic profile with FMRP’s loss of expression. Indeed, metabolomic screenings conducted in *Fmr1* KO mice and patients have shown that glucose, lipid, and several neurotransmitter metabolisms are deregulated in FXS [152,153]. The fact that FMRP binds to the mRNA of many key regulators of metabolism may explain those observations [71,152]. Above all, those results show that the extra-neuronal phenotype caused by FMRP’s absence must not be underestimated and, therefore, deserves to be better characterized.

### 5.1. The Hypocholesterolemic Phenotype of FXS

Cholesterol is a ubiquitous lipid and an essential component of eukaryotic membranes. It is known to play an important role in the maintenance of plasma membranes structural integrity and in the regulation of its functions [154]. Approximately 25% of all cholesterol within human body is found in the brain. Indeed, a large portion of this cholesterol is located in the myelin sheaths of axons and in dendritic spines membranes [155]. Maintenance of cholesterol homeostasis is therefore crucial for proper neurodevelopment and cognitive functions [156].

A prime example of a neurodevelopmental disease caused by an improper cholesterol metabolism is the Smith-Lemli-Opitz syndrome (SLOS), which exhibit mild to moderate ID and ASD as one of its most prevalent comorbidities [157,158]. SLOS is a recessive condition caused by mutations in the gene encoding the 7-dehydrocholesterol reductase, which catalyzes the last step of cholesterol biosynthesis. As such, SLOS patients typically present low levels of circulating cholesterol [158]. A higher prevalence of hypocholesterolemia was also found in an etiologically diverse autistic population [159,160,161]. Moreover, very low levels of circulating cholesterol (below the 10th centiles) were associated with higher risk of ID, anxiety and depression in men with ASD [161].

These observations promoted the investigation of peripheric levels of cholesterol in FXS. Indeed, two independent reports have observed lower plasma levels of LDL (light density lipoprotein), HDL (high-density lipoprotein) and total cholesterol in FXS men [162,163]. Several parameters of those cholesterol profiles were also shown to be correlated with the severity of the behavioral impairments found in these patients. Interestingly, no associations were observed between plasma level of the proprotein convertase subtilisin/kexin type 9 (PCSK9) and total/LDL cholesterol in FXS patients, despite the existence of a clear correlation in control individuals [163]. PCSK9 is secreted from hepatocytes and binds to LDL receptors, which promotes their degradation and favorite higher plasma concentration of cholesterol. Moreover, a recent report showed that the phosphorylation level of PCSK9, a PTM that enhances its binding affinity for the LDL receptor, is decreased in samples from FXS patients [164]. These observations, which suggest a dysregulation of PCSK9 activity in FXS, therefore provides the first clues regarding the molecular mechanism responsible for the hypocholesterolemia found in this population.

Since hypocholesterolemia is a common feature of FXS and ASD, it would be interesting to investigate if the same biochemical mechanisms underlying this phenotype are shared between both conditions. Such research could provide a better understanding of both diseases physiopathology and potentially pave the way for therapeutic approach that could be beneficial for individuals with FXS and ASD. Furthermore, it would also be interesting to validate if low cholesterol level could be used has prognostic marker of ASD and other comorbidities in FXS patients.

### 5.2. Cyclic AMP Metabolism Is Defective in Fragile X

Cyclic AMP (cAMP) is a second messenger involved in the transduction of a plethora of signaling pathways. The intracellular levels of cAMP are regulated by two classes of enzymes: adenylate cyclase (AC) and phosphodiesterase (PDE). Adenylate cyclase is responsible for cAMP synthesis. The majority (9 out of 10) of AC isoforms are membrane-bound enzymes that are regulated by various G protein-coupled receptors (GPCR) [165]. On the other hand, PDE catalyze cyclic nucleotides (cAMP and cGMP) degradation. Some PDE subfamilies are cAMP (PDE4,7 and 8) or cGMP (PDE5,6 and 9) specific, while the other (PDE1,2,3,10 and 11) act on both cyclic nucleotides [166]. Intracellular levels of cAMP promote the activity of various kinases such as PKA and ERK [167] and regulate gene expression through the activation of the transcription factor CREB [165]. As such, cAMP is involved in several cognitive and neuronal processes, including stress, anxiety, memory, synaptic plasticity, synaptic transmission and spine morphogenesis [168,169,170]

The pioneering work of Berry-Kravis et al. in the 1990s provided the first evidence of a defective cyclic AMP metabolism in FXS. Indeed, they reported that blood platelets and lymphoblastoid cell lines from FXS patients produced lower levels of cAMP compared to controls and other matched individuals with ID and ASD [171,172,173]. Furthermore, they reported that FMRP overexpression in mouse neurons enhance cAMP production, thereby highlighting the contribution of FMRP in cAMP pathway [174]. These observations were further replicated in animal models of FXS, more precisely the *Fmr1* KO mouse and the dFMR1 null drosophila [175].

These studies paved the way for the elaboration of the “cAMP theory of fragile X”, which lays its foundation in the aberrant expression pattern of proteins involved in the cAMP cascade. Indeed, mRNAs of several AC isoforms and members of PDE subfamilies are targeted by FMRP [71,121]. This theory further states that the downregulation of cAMP signaling, both downstream and independently of Gp1 mGluR activation, contributes to FXS physiopathology [176]. This theory is corroborated by several studies conducted in KO mice, in which the reversal of several FXS phenotypes was associated with an upregulation of the cAMP pathway. Indeed, pharmacologic treatments that either stimulate adenylate cyclase activity, both directly or through the activation of AC-coupled GPCR or inhibit specific PDE have prove to be efficient therapeutic approach [177,178,179,180].

The accumulation of evidence regarding the defects in the cyclic AMP signaling in FXS and their contribution to the disease physiopathology are undeniable. As such, many effectors of this pathway represent potent therapeutic targets. Furthermore, as shown by the work of Berry-Kravis et al., quantification of cAMP production in peripheral blood cells could serve as objective biomarkers to assay treatment efficacy.

### 5.3. The Amyloid-β Precursor Protein and Its Secreted Metabolites

The Amyloid precursor protein (APP) is a dendritic transmembrane protein which is involved in several neuronal functions, including axonogenesis, neurite outgrowth and neuronal adhesion [181]. APP can be proteolytically processed by two distinct pathways. When undergoing the amyloidogenic pathway, APP is sequentially cleaved by the β-secretase BACE1 and the γ-secretase complex to produce the neurotoxic secreted β-APP (sAPPβ) and the amyloid-β (Aβ) peptides, which either takes the form of a 40 (Aβ40) or 42 (Aβ42) amino acids long chains. Aβ peptides are the major component of the amyloid plaques found in the brain of patients with Alzheimer’s disease and are therefore associated with neurodegeneration. Alternatively, APP can be processed by α-secretases to generate the secreted α-APP (sAPPα), which similarly to APP, bears a wide array of neurotrophic functions [182].

FMRP was shown to bind to APP mRNA and to inhibit its translation through interactions with the miRNA machinery [183]. As such, levels of APP, sAPPα and Aβ peptides were shown to be increased in brains of *Fmr1* KO mice and linked to several key alterations composing the FXS phenotype [184,185]. In accordance with those observations, APP haploinsufficiency was associated with the rescue of repetitive behavior, anxiety, mGluR-LTD and spine morphology in *Fmr1* KO mice [186]. Those alterations regarding APP expression and catabolism are also replicated in humans. Indeed, levels of APP, sAPPα and Aβ peptides were shown to be upregulated in post-mortem brains samples and various peripheral cells from FXS patients [184,186]. Plasma levels of both forms of secreted APP and Aβ peptides were also shown to be increased in children with FXS, while only Aβ42 seems to be decreased in adult patients. Interestingly, circulating levels of secreted APP and Aβ peptides were also shown to be deregulated in children with ASD. Indeed, samples collected from those patients showed an increased in sAPPα and a decreased in sAPPβ and both form of Aβ peptides [187,188,189,190]. Moreover, levels of sAPPα and total sAPP were shown to be reduced following a treatment with acamprosate in children with ASD, both with and without FXS [191].

The dysfunctions regarding APP expression and processing monitored in FXS suggest that those defects could contribute significantly to the mechanisms underlying the disease physiopathology. Furthermore, peripheral levels of APP and its secreted metabolites represent promising biomarkers for the monitoring of treatment efficacy and for the prediction of ASD comorbidities in FXS patients.

## 6. Conclusions

FMRP is a multifunctional protein that plays a role in several cellular and biochemical processes. The phenotypical consequences of its absence are diverse, and many are still misunderstood. Future research should focus on the elucidation of those unanswered questions. Such discovery would promote a better comprehension of the overall mechanism underlying FXS physiopathology and pave the way for new therapeutic approaches and the establishment of objective biomarkers to improve clinical management.

## Data Availability

Not applicable.
